# Abusive supervision and employee voice: The roles of positive reappraisal and employee cynicism

**DOI:** 10.3389/fpsyg.2022.927948

**Published:** 2022-08-09

**Authors:** Wei Sun, Alisher Tohirovich Dedahanov, Abdulkhamid Komil ugli Fayzullaev, Odiljon Sobirovich Abdurazzakov

**Affiliations:** ^1^Management School, Henan University of Urban Construction, Pingdingshan, China; ^2^School of Business, Akfa University, Tashkent, Uzbekistan; ^3^School of Business, Yeungnam University, Gyeongsan, South Korea

**Keywords:** abusive supervision, positive reappraisal, cynicism, promotive voice, prohibitive voice

## Abstract

**Purpose:**

Despite the number of studies on abusive supervision and voice, there is still limited knowledge on why individuals refrain themselves from information sharing. Moreover, very little is known on when individuals become cynical and when they do not under abusive supervision. Hence, to address the existing gaps in the literature this study aims to investigate the moderating role of positive reappraisal on the link between abusive supervision and cynicism; the associations between cynicism and two forms of voice, promotive and prohibitive; and the mediating effect of cynicism on the relationship between abusive supervision and voice.

**Design/methodology/approach:**

We conducted a survey among 685 highly skilled employees and their immediate supervisors in manufacturing companies. Among the 685 responses, we excluded 258 incomplete questionnaires and thus analyzed a total of 427 responses. Hierarchical regression analysis and structural equation modeling were utilized to assess the validity of the hypotheses.

**Findings:**

The findings indicate that positive reappraisal moderates the link between abusive supervision and cynicism; furthermore, cynicism is negatively related to promotive voice and mediates the relationship between abusive supervision and promotive voice. Moreover, the results reveal that the association between cynicism and prohibitive voice is nonsignificant and that cynicism does not mediate the link between abusive supervision and prohibitive voice.

**Originality/value:**

This study is the first to provide empirical evidence on the moderating role of positive reappraisal on the relationship between abusive supervision and cynicism, the association between cynicism and promotive voice and the mediating role of cynicism on the link between abusive supervision and promotive voice.

**Future research directions:**

We recommend that future research consider other forms of voice, such as acquiescent and prosocial voice, in investigating the links between cynicism and employee voice.

## Introduction

Employee voice fosters creativity (Dedahanov et al., 2016a) and increases managerial effectiveness (Morrison, 2011). Moreover, voice helps organizations correct problems (Hirschman, 1970). When individuals refrain from information sharing, their silence can result in corruption (Ashforth and Anand, 2003) and impede innovation (Dedahanov et al., 2016a). Hence, we believe that employee voice has a significant role in business organizations, and it is important to investigate the factors that influence individuals’ intentions to share their opinions and ideas on work-related issues.

Supervision style is perceived to be one of the determinants of employee voice (Dedahanov et al., 2016a). Thus, previous researchers have investigated the link between abusive supervision and employee voice and reported a significant negative link between these two factors (Rafferty and Restubog, 2011; Zhang and Liao, 2015). Despite the existing studies on abusive supervision and employee voice, several gaps exist and need to be addressed.

First, abusive supervisors tend to misbehave by being inconsiderate, publicly criticizing subordinates, and being rude to employees (Bies, 2000; Islam et al., 2021a,b, 2022a). According to James and Shaw (2016), malfeasance generates cynicism that refers to “a general and specific attitude, characterized by frustration and disillusionment as well as negative feelings toward and distrust of a person, group, ideology, social convention, or institution” (Andersson and Bateman, 1997, p.450). Hence, when supervisors ridicule employees and are rude to them, individuals tend to distrust their leadership and become suspicious about supervisors’ intentions behind their supervision in the workplace. Eventually, distrust toward their supervisors makes subordinates reluctant to share their ideas or suggestions to improve the work unit/organization and to speak up on work-related concerns that might cause a serious loss to the work unit. Thus, we believe that cynicism plays a significant role in explaining the link between abusive supervision and employee voice.

Despite the existing research, there is very little knowledge on the role of cynicism in explaining the relationship between abusive supervision and employee voice. Prior research contributed to the literature by investigating the different mechanisms that link abusive supervision and employee voice. For example, previous studies reported that fear (Kiewitz et al., 2016) and perceived injustice (Rafferty and Restubog, 2011; Wang and Jiang, 2015) engendered by abusive supervisors reduce employee voice. We believe that before fearing supervisors and assessing their justice, individuals first consider whether they can trust and rely on their superiors based on their observations. In case of feeling suspicious and experiencing cynicism toward the supervisors, individuals might have a feeling of fear or a perception of injustice because of their distrust of their supervisors’ reactions and treatment. Hence, in our study, we suggest cynicism as a critical mechanism that explains the link between abusive supervision and employee voice.

Second, previous researchers (Seo et al., 2011) contributed to the literature by examining the associations between cynicism and the unitary construct of voice that expresses the frequency of speaking up rather than the multidimensional construct of voice that reflects different types of motives of individuals in voicing their concerns. According to Brinsfield (2013), to fully understand individuals’ information sharing in the workplace, simply examining the frequency of information sharing is insufficient because the unitary construct itself does not express the intentions of individuals to share information. Hence, very little is known about the form of voice that is reduced by cynicism. Thus, in our study, we seek to answer the question regarding which forms of voice are stifled by cynicism by examining the links between cynicism and the multidimensional construct of voice, such as promotive and prohibitive voice.

Third, “abusive supervision may not impact all employees in the same way because the impact of this supervision can vary depending on the abilities of employees in coping with negative events” (Dedahanov et al., 2021, p.468). Thus, researchers (Dedahanov et al., 2022, p.1) indicated that “research has yet to investigate the factors that impact individuals’ response to abusive supervision.” Despite the existing research (Abubakar et al., 2017; Aziz et al., 2017) on the link between abusive supervision cynicism, there is very little knowledge on when individuals experience cynicism under abusive supervision and when they do not. Typically, individuals with positive appraisals perceive negative events to be benign and therefore, tend to reinterpret negative situations positively, which can buffer their negative reactions from perceived abusive supervision. Thus, we believe that even though supervisors are abusive, individuals with positive reappraisal suffer less from the abusive behavior of their superiors; therefore, they are less likely to generate cynicism because of their positive reinterpretation of the negative events. Hence, positive reappraisal can play a contingent role in the link between abusive supervision and cynicism.

To address the existing gaps in the literature, our study aims to examine the associations between cynicism and two forms of voice, promotive and prohibitive, to measure the mediating effect of cynicism on the relationship between abusive supervision and employee voice and to investigate the moderating effect of positive reappraisal on the link between abusive supervision and cynicism.

Our study contributes the literature and extends the insight on the dark side of leadership by enabling the management of organizations to understand why the members of organizations refrain from sharing information under abusive supervision, what type of voice is associated with the cynicism that is triggered by abusive supervision, and when individuals with abusive supervisors experience cynicism and when they do not.

## Employee voice and its multidimensionality

Employee voice is defined as the discretionary communication of suggestions, ideas or concerns about job-related problems to improve the effectiveness of organizational or unit functioning (Morrison, 2011). It is volitional and extra role behavior that is not explained in employees’ job descriptions. There are two constructs of voice: unitary and multidimensional. The unitary construct of voice evaluates voice behavior itself by assessing the frequency of speaking up whereas the multidimensional construct of voice evaluates the intentions of individuals when expressing their opinions. Previous research has suggested several types of multidimensional constructs of voice (Van Dyne et al., 2003; Liang et al., 2012). Van Dyne et al. (2003) suggested acquiescent, defensive and prosocial voice. Acquiescent voice refers to expressing ideas and opinions due to the feeling of resignation, defensive voice is related to sharing ideas to protect and defend oneself from harmful outcomes, and prosocial voice is defined as suggesting constructive solutions with cooperative motives.

Liang et al. (2012) extended the work of previous studies on multidimensionality (Van Dyne et al., 2003; Morrison, 2011) and suggested the concepts of promotive and prohibitive voice. According to Liang et al. (2012), promotive voice refers to “employees” expression of new ideas or suggestions for improving the overall functioning of their work unit or organization” (p. 74). Promotive voice in the workplace relates to employees proactively suggesting new projects that are beneficial to the work unit and voicing their concerns to improve the unit’s working procedures. Thus, Lin and Johnson (2015) indicated that the intention behind promotive voice is to make the organization better. Because promotive voice seeks to improve and change the *status quo*, it is also perceived to be innovative (Lin and Johnson, 2015). Hence, promotive voice reflects the optimistic emotional experiences and eagerness strategy of actors based on approach-oriented self-regulation (Higgins and Spiegel, 2004; Carver and Scheier, 2001; Lanaj et al., 2012).

Prohibitive voice is related to speaking up on work incidents, practices or employee behavior that are perceived as harmful to the organization. According to Liang et al. (2012), individuals with prohibitive voice are more likely to advise others against undesirable behaviors that would hamper job performance and to point out problems when they appear in the unit, even if that would hamper the reporters’ relationships with others. Hence, the intention behind prohibitive voice is to help the company avoid harmful states. Because prohibitive voice draws attention to unnoticed issues and problems, it is perceived to be helpful for groups or organizations as a whole (Lin and Johnson, 2015). Thus, this form of voice focuses on avoidance-oriented tactics and a vigilance strategy (Lanaj et al., 2012).

The aim of both types of voice is to benefit the organization and challenge the *status quo* (Liang et al., 2012). However, promotive and prohibitive voices differ in terms of their behavioral content, function, and implications for others. According to Liang et al. (2012), promotive voice expresses new solutions and ideas about how to advance the *status quo* whereas prohibitive voice conveys concerns about factors that are perceived as threatening to the organization. Moreover, promotive voice is future-oriented whereas prohibitive voice is oriented to both the past and the future. Furthermore, promotive voice suggests methods to improve an organization whereas prohibitive voice aims to prevent the factors that are potentially harmful to an organization.

## Abusive supervision and cynicism

Abusive supervisors’ behavior includes derogatory language, explosive outbursts and threatening statements (Kuo et al., 2015). When supervisors are rude to employees, they develop an unhealthy work environment where individuals lose their trust, loyalty and energy toward not only the inflicting supervisor but also the organization or management for taking a “hands-off” approach to abusive supervisors (Li et al., 2016; Abubakar et al., 2017).

Similarly, Tepper et al. (2004) stated that when supervisors are abusive, individuals distrust their supervisor’s leadership and work-related instructions. According to Li et al. (2016), cynical individuals do not trust superiors and are suspicious about management. Hence, abusive supervision shapes cynicism by reducing trust from subordinates. In other words, when supervisors ridicule subordinates and express anger, employees tend to lose their hope for the future in current organizations and are more likely to distrust their management.

Previous studies also indicated that there is a positive link between the abusive behavior of supervisors and cynicism (Abubakar et al., 2017; Aziz et al., 2017). Aziz et al. (2017) stated that abusive supervisors insult employees and view them inferior, eventually triggering employees’ cynicism in the workplace. Keashly (1998) indicated that cynicism is one of the consequences of abusive supervision. Thus, in line with previous findings, we also believe that abusive supervision fosters cynicism and propose the following hypothesis:

**H1:** *Abusive supervision is positively related to cynicism.*

## Moderating role of positive reappraisal

A number of previous studies have indicated that abusive supervision triggers cynicism (Keashly, 1998; Whitman et al., 2014). According to Chi and Liang (2013), the impact of abusive supervision on individuals differs depending on the capabilities of individuals to handle negative experiences. Individuals with positive reappraisal perceive negative and stressful events as valuable, benign, and beneficial (Saintives and Lunardo, 2016). Moreover, individuals with positive reappraisal may reframe a negative event in workplaces as an opportunity to gain new skills (Lambert et al., 2012). Consequently, when employees reconstrue negative events positively, they can buffer against abusive supervision generating negative views toward management or organization (Chi and Liang, 2013).

Hence, we believe that not everyone who experiences abusive behavior from superiors becomes cynical. Those who have positive reappraisal are less likely to suffer from cynicism despite the abusive behaviors of their superiors. In contrast, when individuals do not modify their thoughts about workplace abuse, they are more likely to become cynical toward their employers. Therefore, we suggest that positive reappraisal weakens the relationship between abusive supervision and cynicism. In other words, even though supervisors are rude to employees, individuals with positive reappraisal tend to reconstrue their thoughts about this unpleasant behavior of their superiors. Eventually, individuals with positive reappraisal are less likely to lose their hope or trust in their superiors. Thus, based on the discussions above, we propose the following hypothesis:

**H2:** *Positive reappraisal moderates the relationship between abusive supervision and cynicism such that the positive relationship is weaker when the level of positive reappraisal is high.*

## Cynicism and employee voice

Cynics tend to be suspicious about the statements of their superiors (Kanter and Mirvis, 1989); therefore, employees with cynicism distrust the strategies of their managers and doubt the intentions of their management (Stanley et al., 2005). Similarly, Wanous et al. (2000) indicated that individuals with cynicism do not trust their management and thus perceive that sharing opinions and ideas about work is worthless because it cannot be heard by superiors. When individuals distrust management, perceive that their attempts to make improvements would not be recognized and personal initiative does not count for much in their workplace, they become reluctant to put effort into sharing ideas that are beneficial to the work unit/organization and do not raise suggestions to improve their unit’s/organization’s working procedures. Moreover, when individuals become cynical, they tend to develop withdrawal behaviors (Abubakar et al., 2017), which are associated with avoiding work roles and reducing the time spent on work (Wang and Yi, 2012). Further, cynical individuals tend to have lower levels of organizational commitment and are more likely to be absent from their work (Seo et al., 2011). Furthermore, individuals with cynicism tend to lose their motivation in their given roles and tasks (Reichers et al., 1997). When individuals withdraw from their work, have a lower level of commitment, and have reduced workplace motivation, they feel a lower level of emotional attachment to their work. Hence, because of their lower level of emotional attachment, cynical individuals are less likely to consider improving their unit/organization and to share constructive suggestions that help the unit/organization reach its goals. Therefore, we believe that cynicism negatively influences employees’ promotive voice.

**H3:** *Cynicism is negatively related to promotive voice*

Furthermore, cynicism is related to a number of negative emotions. According to Dean et al. (1998), one of those emotions is fear. When individuals disbelieve the intentions of their superiors, they do not feel safe in expressing work-related concerns because they may be punished or threatened (Dedahanov and Rhee, 2015). According to Liang et al. (2012), the intention behind promotive voice is understood as positive and constructive; however, the intention behind prohibitive voice may not be regarded as positive because of the negative consequences that can occur as a result of avoidance-oriented expression. Pointing out a flaw or defect to supervisors entails a risk of damaging or even humiliating other organizational members because such voice can reveal their mistakes or unwise judgements. Such a suggestion can trigger self-defense by listeners, and listeners may retaliate against the idea provider uncovering their myopia. Since having prohibitive voice can trigger negative reactions, cynical individuals tend to assess the consequences of speaking up (e.g., retaliation by receptors) prior to sharing the information. Therefore, we believe that cynics are less likely to have prohibitive voice by refraining themselves from speaking up about work-related problems that might cause serious losses to the work unit/organization because of self-defense. Thus, based on the discussions above, we propose the following hypothesis.

**H4:** *Cynicism is negatively related to prohibitive voice.*

## Mediating role of cynicism

Previous studies (Rafferty and Restubog, 2011; Zhang and Liao, 2015) indicated that abusive supervision mitigates employee voice. We believe that this relationship between abusive supervision and employee voice is mediated by cynicism. In other words, when supervisors mistreat employees and ridicule them, individuals tend to have a lower level of trust in their superiors and are more likely to lose their hope for the future. Consequently, with the reduced trust in superiors, individuals are less likely to suggest their concerns regarding the improvement of unit/organization because of their belief that speaking up would not result in any change in their workplace. Hence, we assume over a night individuals do not decide to withhold information that makes a change in the workplace rather they first carefully observe the behaviors of superiors and in case of experiencing abuse from superiors they might lose their trust and become cynic which eventually reduces their promotive voice.

Moreover, abusive supervisors give high signals of threat that leads individuals to use self-protection and avoidance which eventually fosters individuals’ defensive silence (Kiewitz et al., 2016) and reduced prohibitive voice. We believe that when individuals suffer the mistreatment from superiors they become cynical and might be concerned with the reactions of their superiors for having prohibitive voice because prohibitive voice can be interpreted negatively by superiors and therefore can trigger negative interpersonal outcomes. Thus, individuals do not decide to have prohibitive voice all of sudden, rather they first become cynical and because of their cynicism in the reactions of their superiors they tend to have reduced prohibitive voice. Hence, we believe that cynicism can be a critical mechanism that explains the link between abusive supervision and employee voice and suggest the following hypotheses:

**H5:** *Cynicism mediates the link between abusive supervision and promotive voice.*

**H6:** *Cynicism mediates the link between abusive supervision and prohibitive voice.*

## Materials and methods

We conducted survey in various manufacturing sector companies located in five cities of Republic of Korea (i.e., Daegu, Incheon, Busan, Ulsan, and Daejon). We contacted the human resource management departments of 17 manufacturing companies and explained the purpose of our study. With the help of the human resource managers of the companies, we obtained a list of 685 employees and their immediate supervisors. We collected data using convenience sampling procedure (Islam et al., 2022b). We used two waves of data collection to reduce the effect of common method bias (Podsakoff et al., 2003). The first-wave survey was conducted among employees. Paper-and-pencil surveys were conducted in small group sessions. Participating in the survey was voluntary and the respondents were provided verbal and written assurance of the confidentiality of responses. Employees answered questions pertaining to abusive supervision, positive reappraisal and cynicism. Supervisors answered the questions regarding employee voice. The survey questionnaires were coded before conducting the survey, and HR managers helped record identification numbers and the participants’ names to match supervisor–subordinate dyads. We deleted 258 carelessly completed questionnaires and used 427 questionnaires in the analyses (the response rate was 62%).

### Measures

The questionnaires were translated from English into Korean by professional translators, and bilingual experts back-translated the questions into English to ensure the accuracy of the translation (Brislin, 1993). In our study, all measures were rated on a five-point Likert scale ranging from “1 = strongly disagree” to “5 = strongly agree.”

**Abusive supervision** was measured using 13 items (e.g., “My supervisor ridicules me,” “My supervisor puts me down in front of others,” and “My supervisor is rude to me”) from Tepper (2000). The scale’s α reliability value in this study was 0.982.

**Positive reappraisal** was measured using 4 items from the study by Carver et al. (1989). Example items included: “I look for something good in what is happening,” “I try to see it in a different light to make it seem more positive” and “I learn something from experiences.” The Cronbach’s α reliability in this study was 0.855.

**Cynicism** was assessed using 11 items (e.g., “When top management says it is going to do something, I wonder if it will truly happen,” “I believe top management says one thing and does another” and “When I think about top management, I feel irritated”) from the study by Kim et al. (2009). The scale’s α reliability in this study was 0.880

**Employee voice:** Promotive voice (e.g., “ This employee proactively suggests new projects that are beneficial to the work unit,” “This employee proactively develops and makes suggestions for issues that may influence the unit” and “This employee raises suggestions to improve the unit’s working procedures”) was evaluated using 5 items, and prohibitive voice (e.g., “This employee advises others against undesirable behaviors that would hamper job performance,” “This employee speaks up honestly with problems that might cause serious losses to the work unit, even when/though dissenting opinions exist” and “This employee dares to voice opinions on things that might affect efficiency in the work unit, even if it would embarrass others”) was assessed with 5 items from the study by Liang et al. (2012). The scales’ α reliability values in this study were 0.948 and 0.890, respectively.

Because of the potential impacts of age, gender (0 = female and 1 = male) and organizational tenure (1 = 5 years or less, 2 = 6–10 years, 3 = 11–15 years, 4 = 16–20 years, and 5 = more than 20 years) of respondents on employee voice, we used these sample characteristics as control variables in our study (Table 1).

**TABLE 1:** Demographic characteristics of participants.

Respondents	Gender		Age				Work experience				
			
	Male	Female	25–35 years old	36–45 years old	46–55 years old	56–65 years old	5 years	6–10 years	11–15 years	16–20 years	More than 20 years
Supervisors	81.1%	18.9%	7.5%	50.9%	35.8%	5.7%	7.5%	30.2%	39.6%	17.0%	5.7%
Employees	59.7%	40.3%	19.2%	42.9%	31.8%	6.1%	22.5%	49.9%	23.2%	3.3%	1.2%
											

## Results

In our study, to evaluate the measurement model, we conducted confirmatory factor analysis (CFA) by using AMOS 21. Researchers (Hooper et al., 2008; Kline, 2010) recommended reporting χ2-test goodness-of-fit indices, including the comparative fit index (CFI), goodness-of-fit index (GFI), adjusted goodness-of-fit index (AGFI), root mean square residual (RMR), root mean square error of approximation (RMSEA) and standardized root mean square residual (SRMR). A good model fit is indicated when the CFI and GFI values exceed 0.90 (Hair et al., 2010) and the RMSEA and SRMR values are less than 0.60 and 0.05, respectively (Hair et al., 2010). In our study, all measures indicated a good fit to the data (χ2/df = 1.507; CFI = 0.985; GFI = 0.899; AGFI = 0.880; RMR = 0.051 RMSEA = 0.034, and SRMR = 0.0254).

Moreover, we assessed the convergent and discriminant validity. Convergent validity is demonstrated when composite reliabilities and factor loadings are greater than 0.80 and 0.60, respectively (Fornell and Larcker, 1981). The findings demonstrate that composite reliabilities and factor loadings exceeded the required thresholds of 0.80 and 0.60, respectively.

Discriminant validity is demonstrated when the average variance extracted (AVE) values of constructs exceed 0.5 and the squared correlation between the same construct and other constructs (Hair et al., 2010). The results indicate that all AVEs of constructs are higher than 0.5 and the squared correlations between the construct and other constructs in the CFA model (see Table 2). Thus, the measures demonstrate discriminant validity.

**TABLE 2:** Descriptive statistics, average variance extracted (AVEs), correlations and internal consistency reliabilities.

Variables		Mean	SD	AVE	1	2	3	4	5
1	Abusive supervision	3.145	1.291	0.804	1				
2	Positive reappraisal	2.885	1.388	0.844	–0.144**	1			
3	Cynicism	3.218	1.299	0.816	0.150**	–0.496**	1		
4	Promotive voice	2.977	1.256	0.775	–0.114*	0.097*	–0.184**	1	
5	Prohibitive voice	3.078	1.289	0.795	–0.062	0.077	–0.076	–0.001	1

*P < 0.05 and **P < 0.01.

The findings from the correlation analysis (Table 2) reveal that positive reappraisal is negatively correlated with cynicism (*r* = –0.496, *p* < 0.01) and cynicism is positively correlated with abusive supervision (*r* = 0.150, *p* < 0.01) and negatively correlated with promotive voice (*r* = –0.184, *p* < 0.01).

**FIGURE 1:**
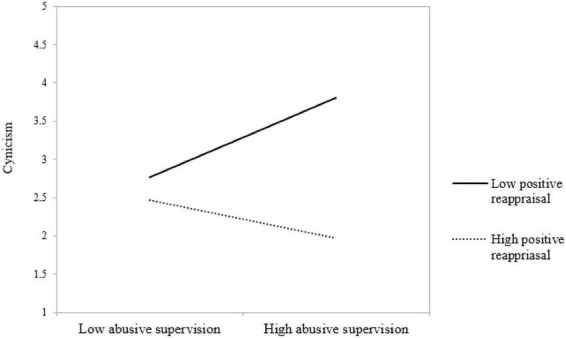
Moderating effect of positive reappraisal on the relationship between abusive supervision and cynicism.

To evaluate the validity of the suggested hypotheses, we conducted structural equation modeling (SEM) and hierarchical regression analysis. The SEM analysis indicates a good fit (χ2/df = 1.598; CFI = 0.982; GFI = 0.895; AGFI = 0.876; RMR = 0.072, RMSEA = 0.037, and SRMR = 0.0412). The findings from SEM analysis suggest that abusive supervision (β = 0.146, *p* < 0.01) is negatively associated with cynicism. Thus, H1 is supported. In H2, we suggested that positive reappraisal moderates the link between abusive supervision and cynicism. Hierarchical regression analysis revealed that positive reappraisal moderates (β = –0.195, *p* < 0.01) the link between abusive supervision and cynicism (Table 3). Moreover, we plotted the simple slopes for the relationship between abusive supervision and cynicism to interpret the form of the interaction (see Figure 1). When individuals have a higher level of positive reappraisal, abusive supervision is associated with a lower level of cynicism (β = –0.168, *p* < 0.01). When individuals have a lower level of positive reappraisal, simple slopes demonstrate that abusive supervision is associated with a higher level of cynicism (β = 0.369, *p* < 0.01). Hence, H2 is also supported.

**TABLE 3:** Results of hierarchical moderated regression analyses for positive reappraisal.

	Model 1	Model 2	Model 3
Age	0.050	0.042	0.049
Work experience	–0.052	–0.042	–0.049
Gender	0.025	0.014	–0.019
Abusive supervision		0.079	0.735*
Positive reappraisal		–0.452*	0.197
Ab. Supervision X Pos. Reappraisal.			–0.195*
*R* ^2^	0.002	0.253	0.323
Adjusted *R*^2^	–0.005	0.244	0.314
Change in *R*^2^		0.251	0.07
F	0.250	28.520	33.425

Dependent variable: Cynicism. *p < .01.

Moreover, the findings suggest that cynicism is negatively and significantly related to promotive voice (β = –0.201, *p* < 0.01). Thus, H3 is also supported. Further, the findings reveal that the link between cynicism and prohibitive voice (β = –0.079, *p* > 0.05) is nonsignificant. Hence, H4 is not empirically supported. To assess the mediating role of cynicism on the link between abusive supervision and voice, we followed the suggestions of Preacher and Hayes (2008) by using a bootstrapping procedure. By extracting 1,000 bootstrapped samples from the dataset based on random sampling with replacement, 95% bias-corrected confidence intervals (CIs) were calculated. If the CI of an indirect effect did not include 0, mediation was assumed (Lichtenthaler and Fischbach, 2016). Bootstrapping analysis indicates that cynicism mediates the link between abusive supervision and promotive voice (β = –0.029, *p* < 0.01; CI_0.95_ = –0.061, –0.036) and does not explain the link between abusive supervision and prohibitive voice (β = –0.012, *p* > 0.05; CI_0.95_ = –0.009, 0.002). Therefore, H5 is supported but H6 is not supported.

## Discussion

This study investigated the contingency role of positive reappraisal on the link between abusive supervision and cynicism; the relationships between cynicism and two forms of voice, promotive and prohibitive; and the mediating effect of cynicism on the link between abusive supervision and voice (Table 4).

**TABLE 4:** Results of mediation analysis.

	*p* value	Standardized coefficient
**Indirect effects**		
Abusive supervision → Cynicism → Promotive voice	0.001	–0.029*
Abusive supervision → Cynicism → Prohibitive voice	0.143	–0.012

*p < .01.

The findings revealed that positive reappraisal moderates the link between abusive supervision and cynicism. In other words, when supervisors are rude and abusive, individuals who look for good in what occurs and who modify their thoughts about negative experiences are less likely to doubt the intentions of superiors by having a lower level of cynicism. In contrast, when supervisors are abusive, individuals who do not reinterpret negative situations and experiences positively tend to be cynical. Hence, the higher the positive reappraisal is, the weaker the relationships between abusive supervision and cynicism will be. The lower the positive reappraisal is, the stronger the link between abusive supervision and cynicism.

Moreover, the results suggest that cynicism is negatively related to promotive voice. That is, when individuals are suspicious about the motives of superiors and do not trust them, they are less likely to suggest recommendations on improving their work unit/organization. Based on this finding, we could confirm that cynical individuals tend to perceive that sharing ideas to improve a work unit/organization might be futile and does not result in any change in the organization because of their doubts regarding the intentions and goals of superiors; therefore, they prefer to refrain from expressing their work-related concerns. This finding is consistent with the statement of Wanous et al. (2000) who posited that cynics tend to perceive that sharing work-related concerns is useless because it cannot be heard by management. Contrary to our expectation, the results indicate that there is no significant relationship between cynicism and prohibitive voice. This nonsignificant link between cynicism and prohibitive voice can be explained in the following way. Some work-related problems that are harmful to a unit/organization can also negatively impact employees by reducing their work hours and salaries and pose a threat to their careers. Hence, to protect themselves from the undesirable outcomes of work-related problems, some cynics can share work-related issues that may have a direct impact on them without any fear and hesitation while other members with cynicism may remain silent because of their expectation of potential harm from superiors due to sharing information rather than the direct impact of work-related problems on their careers.

Further, the findings reveal that cynicism mediates the relationship between abusive supervision and does not explain the links between abusive supervision and prohibitive voice. That is, when supervisor ridicule and are rude to employees, individuals become cynic which eventually inhibit their intention to share their concerns on improving the organization’s/unit’s working procedure. Thus, we believe that individuals suffering from abusive supervision do not decide to refrain themselves on work related issues overnight, rather they first lose their trust and become cynic and eventually give up speaking up on work-related issue because of their perception that speaking up does not change the situation.

### Theoretical implication

Our findings contribute to the literature in several ways. First, previous studies (Abubakar et al., 2017; Aziz et al., 2017) on abusive supervision reported that abusive supervision fosters cynicism among the members of organizations. Our study suggests that not all individuals working under abusive supervision experience cynicism. Individuals who reinterpret negative situations positively are less likely to become cynical despite the abusive behavior of superiors. Hence, this study enriches the abusive supervision literature by investigating the boundary condition role of positive reappraisal on the link between abusive supervision and cynicism.

Second, prior research (Seo et al., 2011) that examined the association between cynicism and voice mainly focused on the unitary construct of voice rather than the multidimensional construct.

We believe that evaluating the frequency of speaking up is insufficient because voice behavior itself does not express the motives of employees in voicing their concerns. Hence, our study contributes to the literature by providing a richer understanding of the form of voice that is engendered by cynicism.

Third, despite the extensive study on abusive supervision and employee voice, there was a lack of knowledge on why individuals under abusive supervision refrain themselves from information sharing. As mentioned earlier, prior research reported fear and a perception of injustice as the mechanisms that link abusive supervision and employee voice; however, we believe that individuals do not generate a fear and a perception of injustice and make up their mind to give up voicing overnight; rather, they first assess the behaviors of supervisors and become cynical by distrusting and doubting the plans and intentions of superiors. Consequently, as the perception of speaking up does not make any difference, individuals become reluctant to share their work-related concerns on improving the workplace. Thus, our study extends the literature by suggesting cynicism as a crucial mechanism that explains the link between abusive supervision and promotive voice.

## Practical implications

The findings suggest that abusive supervision fosters cynicism. We recommend that organizations give clear guidelines to individuals about the punishment for defying organizational policies and rules by using appropriate punishment systems (Robinson and O’Leary-Kelly, 1998). Using appropriate punishment systems can reduce the misconduct and prohibited activities of superiors.

Moreover, to reduce cynicism, supervisors should develop trustworthiness by establishing supportive and fair cultures in organizations. Further, honest and open communication between supervisors and employees also reduces cynicism (Li and Chen, 2018) because when employees have open communication with their superiors, they are more likely to know the real intentions and goals of their supervisors, which consequently reduces their doubt and disbelief.

Furthermore, supervisors should make individuals feel that they play important roles in developing the organization. Superiors can give this perception to employees by listening and encouraging them to suggest new ideas that improve the work environment and allow individuals to participate in decision-making processes. By doing so, organizations can mitigate the cynical perceptions of employees and foster employee voice (Abubakar et al., 2017).

Furthermore, our study reveals that positive reappraisal weakens the link between abusive supervision and cynicism. Hence, we suggest that organizations offer training programs that educate employees how to control their thoughts in critical situations and increase their emotional intelligence.

## Limitations and future research directions

Despite the fact that our study extends the literature, it also has several limitations that should be addressed by future studies. First, our study investigates the associations between cynicism and two forms of voice: promotive and prohibitive. We recommend that future research consider other forms of voice, such as acquiescent and prosocial voice, in investigating the links between cynicism and employee voice.

Second, in this study, we used the age, gender and work experience of individuals as control variables. Since individuals’ cultural values can also influence their voice behavior (Dedahanov et al., 2016b), we recommend that future research investigate cultural factors as control variables. Third, we conducted the study in one country, the Republic of Korea; thus, the generalizability of the findings can be another limitation. Therefore, to yield more generalizable results, this type of research should be conducted in several countries. Fourth, our study utilized a cross-sectional design that evaluates the correlational mechanisms rather than the dynamic aspects of the determinants of voice. Thus, we recommend that future research utilize a longitudinal design.

## Conclusion

This study examined the contingency role of positive reappraisal on the association between abusive supervision and cynicism; the link between cynicism and two types of voice namely, promotive and prohibitive and the role of cynicism in explaining the link between abusive supervision and voice. Questionnaires collected from 427 respondents were analyzed in this study. We used hierarchical regression analysis and structural equation modeling to assess the validity of proposed hypotheses. The results suggest that positive reappraisal moderates the association between abusive supervision and cynicism; cynicism mitigates promotive voice and explains the link between abusive supervision and promotive voice. Further, the findings suggest that the link between cynicism and prohibitive voice is not significant and cynicism does not play a mediating role between abusive supervision and prohibitive voice. Our study extends the literature by providing first empirical evidence on the contingency role of positive reappraisal on the link between abusive supervision and cynicism, the association between cynicism and promotive voice and the mediating role of cynicism on the association between abusive supervision and promotive voice.

## Data availability statement

The raw data supporting the conclusions of this article will be made available by the authors, without undue reservation.

## Ethics statement

Ethical review and approval was not required for the study on human participants in accordance with the local legislation and institutional requirements. Written informed consent from the patients/participants or patients/participants legal guardian/next of kin was not required to participate in this study in accordance with the national legislation and the institutional requirements.

## Author contributions

WS designed the research conceptual model, wrote the theoretical background, and acquired the funding. AD wrote hypotheses, theoretical and practical implications section and supervised the project. AF conducted a survey, coded the data and analyzed the survey data. OA analyzed the data, interpreted the results, wrote the discussion part, reviewed and edited the manuscript. All authors contributed to the article and approved the submitted version.
